# Development of gut mycobiome in infants and young children: a prospective cohort study

**DOI:** 10.1038/s41390-023-02471-y

**Published:** 2023-01-20

**Authors:** Jenni Turunen, Niko Paalanne, Justus Reunanen, Terhi Tapiainen, Mysore V. Tejesvi

**Affiliations:** 1 Research Unit of Clinical Medicine, University of Oulu, Oulu, Finland; 2 Biocenter Oulu, University of Oulu, Oulu, Finland; 3 Department of Pediatrics and Adolescent Medicine, Oulu University Hospital, Oulu, Finland; 4 Research Unit of Translational Medicine, University of Oulu, Oulu, Finland; 5 Ecology and Genetics, Faculty of Science, University of Oulu, Oulu, Finland

## Abstract

**Background:**

The composition of the gut fungal microbiome, mycobiome, is likely associated with human health. Yet, the development of gut mycobiome is poorly understood in infants and children. Here we investigate how perinatal events influence the development of gut mycobiome.

**Methods:**

In this prospective cohort study of 140 infants, we used ITS gene sequencing of fecal samples from birth to the age of 18 months. We compared gut mycobiome composition according to delivery mode and exposure to intrapartum antibiotics during vaginal delivery.

**Results:**

At birth, gut mycobiome were dominated by the genus *Candida*, at 6-month stool samples by *Malassezia* and *Cystofilobasidium*, and the 18-month stool samples by *Trichosporon* and unidentified fungi. Perinatal factors altered mycobiome. At 18 months, gut mycobiome of infants born vaginally consisted mostly of *Trichosporon* (32%) and unidentified fungi (31%), while those born via Cesarean section delivery samples had mycobiome dominated by *Saccharomyces* (50%). At the age of 18 months, those exposed to intrapartum antibiotics had mycobiome dominated by *Trichosporon* (66%) not seen in those unexposed to antibiotics.

**Conclusions:**

Delivery mode and exposure to intrapartum antibiotic prophylaxis were markedly associated with gut mycobiome composition from birth to 18 months of age.

**Impact:**

- The composition of the gut mycobiome is likely associated with human health. Yet, the development of gut mycobiome is poorly understood in infants and children.
- In this prospective cohort study, delivery mode and exposure to intrapartum antibiotic prophylaxis were markedly associated with gut mycobiome composition from birth to 18 months of age.
- The impact of intrapartum antibiotic prophylaxis on fungal microbiome in vaginally born infants, previously shown to influence gut bacteriome composition, may be explained by the interaction between bacteria and fungi.
- Gut mycobiome composition likely deserves further investigation in relation to gut microbiome and health in children.

## Introduction

The gut microbiome consists of bacteria, viruses, fungi, archaea, and protozoa, but while the human gut microbiome has been studied extensively, there are limited data available that extend beyond the bacterial microbiome, also known as the bacteriome. It is only recently that the number of studies of the gut mycobiome, i.e. fungi in the gut, has started to increase regarding both animals^1–4^ and humans.^1,4–7^

The composition of the gut mycobiome has been shown to be associated with human health. Decreased biodiversity and a higher prevalence of the genera *Candida* and *Malassezia*, as well as strong bacteria-fungi associations, have been linked with inflammatory bowel disease (IBD)^8,9^ and irritable bowel syndrome (IBS),^8,10^ and the gut mycobiome has been associated with obesity, particularly with fungi related to lipid and glucose metabolism.^11^ The presence of *Candida* in the gut microbiome has been thought to contribute to glucose metabolism disorders,^12^ and shifts in the gut mycobiome have also been associated with diseases, such as colorectal cancer,^13^ chronic liver diseases,^14^ atopic dermatitis,^15^ and multiple sclerosis.^16^ Fungal colonization of the gut likely starts at birth, at least partially through vertical transmission from mother to child.^17,18^ Willis et al. studied the presence of fungi in the feces of newborn infants and found a low biomass mycobiome.^19^ In preterm infants, mycobiome dominated by the genus *Candida* has been observed.^20^ There are limited data on early-life factors affecting gut mycobiome composition. We have shown previously that exposure to antibiotics at birth markedly changes the composition of the gut bacteriome^21^ and its main source shift from mother to other sources.^22^

We set out here to characterize the development of the gut mycobiome in a prospective cohort study of 140 newborn infants from birth to 18 months of age.

## Materials and methods

### Study design and population

This was a prospective cohort study of 140 newborn infants investigating the impact of early-life factors on the development of the gut mycobiome from birth to 18 months of age. The protocol was reviewed and found acceptable by Ethical Committee of Northern Ostrobothnia Hospital District at Oulu University Hospital, Finland, decision number 3/2016. The families gave their written informed consent in advance, and the study was conducted according to the relevant guidelines and restrictions for clinical research. Altogether 434 fecal samples were collected (Table 1), and 50 negative control samples (HyClone™ HyPure, Thermo Fisher Scientific, Waltham, MA) were prepared together with the fecal samples for contamination removal.

**Table 1:** Fecal samples collected for the study by sampling time, delivery mode, and exposure to intrapartum antibiotics at birth (first-pass meconium, 6-month stool, 18-month stool) and by delivery mode and antibiotic usage (vaginal delivery, vaginal delivery with intrapartum antibiotics, C-section delivery with intrapartum antibiotics).

	At birth	At 6 months	At 18 months
Vaginal delivery	56	59	59
Vaginal delivery with intrapartum antibiotics	24	33	23
C-section delivery with intrapartum antibiotics	60	60	60

### Sample collection

Meconium samples were obtained from a diaper by a midwife in the delivery room or by the nurse responsible for the child in the labor ward. The infant stool samples at 6 and 18 months were collected by the families at home. All the samples were stored at −80 °C until further processing.

### DNA extraction

DNA was extracted from the meconium and stool samples using the DNeasy PowerSoil Pro kit (Qiagen, Hilden, Germany) according to the manufacturer’s protocol. In all, 200 mg of sample was weighed out, and 1 ml of phosphate-buffered saline was added to each sample. The samples were homogenized by bead beating with Tissuelyzer (Qiagen) for 2 min at 25 Hz and incubated in ice for 1 min. This homogenization was repeated 1–3 times. The negative control samples and meconium samples with little material were not put through the Tissuelyzer homogenization, but instead the samples followed the protocol for vortex adapter homogenization. After homogenization, extraction was performed on a QIAcube Connect extraction machine (Qiagen) with the final elution set to 100 µl. The quantity and quality of the DNA were measured using a NanoDrop 1000 Spectrophotometer (Thermo Fisher Scientific).

### PCR, sequencing, and analysis

Sequencing of the internal transcribed spacer 2 (ITS2) gene was performed using the primer fITS7b (5’-GTGARTCATCGAATCTTTG-3’) and the primers ITS4 (5’-TCCTCCGCTTATTGATATGC-3’) with unique barcodes. PCR was conducted using the Phusion Flash High-Fidelity PCR master mix (Thermo Fisher Scientific) according to the manufacturer’s protocol. An additional negative control (sterile water, HyClone™ HyPure, Thermo Fisher Scientific) and a positive control, Mycobiome Genomic DNA Mix (msa-1010, ATCC, VA) were added to each PCR plate. The reactions were performed with an Applied Biosystems™ Veriti 96-Well Thermal Cycler machine (Thermo Fisher Scientific). The PCR program started with 2 min of initialization, followed by 35 cycles of denaturation at 98 °C for 10 s, annealing at 54 °C for 20 s and elongation at 72 °C for 30 s. The final elongation at 72 °C lasted for 7 min. The PCR products were imaged by agarose gel electrophoresis in 1.5% TAE agarose gel with ethidium bromide dye using a VersaDoc (Bio-Rad, Hercules, CA) imaging machine.

Sequencing was performed on an IonTorrent PGM platform using a method described previously,^23^ and the sequence data were analyzed using the QIIME2 platform (version 2021.2).^24^ Sequences less than 200 bp long were omitted from the analysis. QIIME2-implemented DADA2 was used to demultiplex and denoise the sequence data.^25^ Reads were trimmed at 15 and truncated at 160, and chimeric reads were filtered out. As microbiome studies are sensitive to contamination,^26^ we removed instances of environmental contamination from the samples with the R package decontam (version 1.14.0) using 50 negative controls (sterile water), using a prevalence-based method with a threshold of 0.5.^27^

To calculate the within-sample diversity or alpha diversity, we rarefied the samples at 1004 and used the Shannon Index and observed features as metrics, with Kruskal–Wallis *H* as the statistical test. Between-sample diversity, or beta diversity, was calculated using Principal Coordinate Analysis with Bray–Curtis Dissimilarity. PERMANOVA was used to measure the statistical significance of these results. All the statistical tests were further evaluated with a *p* value, for which 0.05 was considered statistically significant. For alpha diversity tests, we used a *p* value with a Benjamini–Hochberg false discovery rate. We used the UNITE database (version 8.3) for the taxonomic analysis,^28^ and analysis of composition of microbiomes (ANCOM)^29^ together with the Mann–Whitney *U*-test to analyze the statistical significance of the taxonomic differences between the sample groups. The images were produced using Rstudio (R version 4.2.2) with the package ggplot2 (version 3.4.0), and the panel images were finalized with Inkscape (version 1.1). The raw sequences were uploaded in BioProject with the accession number PRJNA831656.

## Results

We enrolled 56 infants born vaginally, 24 born vaginally and exposed to intrapartum antibiotics, and 60 born via Cesarean section. The characteristics of the cohort are presented in Table 2.

**Table 2:** Population characteristics of the participating infants (*n* = 140).

	Vaginal delivery *n* = 56	Vaginal delivery with antibiotics *n* = 24	C-section with antibiotics *n* = 60
Maternal characteristics			
Maternal age (years), mean (SD)	29.7 (5.4)	28.5 (5.2)	31.6 (6.5)
Number of siblings, mean (SD)	1.5 (2.6)	1.0 (1.7)	1.3 (1.4)
Maternal asthma, *N* (%)	5 (8.9)	1 (4.2)	11 (18.3)
Maternal allergy, *N* (%)	14 (25.0)	5 (20.8)	20 (33.3)
GDM	5 (8.9)	6 (25.0)	16 (26.7)
Smoking during pregnancy	3 (5.4)	2 (8.3)	12 (20.0)
* Str. agalactiae* positive^a^	1 (1.8)	21 (87.5)	9 (15.0)
Antibiotics during pregnancy^b^	8 (14.3)	12 (50)	16 (26.7)
Newborn characteristics			
Female (%)	24 (42.9)	10 (41.7)	29 (48.3)
Gestational age (weeks), mean (SD)	39.9 (1.1)	39.9 (1.3)	38.9 (1.3)
Birth weight (g), mean (SD)	3480 (360)	3540 (550)	3490 (610)
Apgar 1 min, mean (SD)	8.8 (1.0)	8.3 (1.6)	8.7 (1.0)
Apgar 5 min, mean (SD)	9.2 (0.8)	9.0 (0.9)	9.1 (0.6)
Apgar 15 min, mean (SD)	9.5 (0.6)	9.2 (0.9)	9.4 (0.5)
Perinatal antibiotics, *N* (%)^c^	0	1 (4.2)	1 (1.7)

*GDM* gestational diabetes mellitus.

^a^Maternal *S. agalactiae* screening was not performed for 24 mothers in the C-section group.

^b^One mother in the vaginal delivery group had received cephalexin, 1 amoxicillin, 1 nitrofurantoin, 1 pivmecillinam, and 1 topical metronidatzole. For 4 mothers, the antibiotic used was not recorded. Two mothers in the vaginal delivery with antibiotics group had received pivmecillinam and 1 had received amoxicillin, but the antibiotic used was not recorded for 9 mothers. Two mothers in the C-section group had received cephalexin, 2 amoxicillin, 2 topical metronidatzole, 1 nitrofurantoin, and 1 clindamycin. For 8 mothers, the antibiotic had not been recorded.

^c^Both children received a combination of benzyl penicillin and tobramycin.

**Fig. 1:**
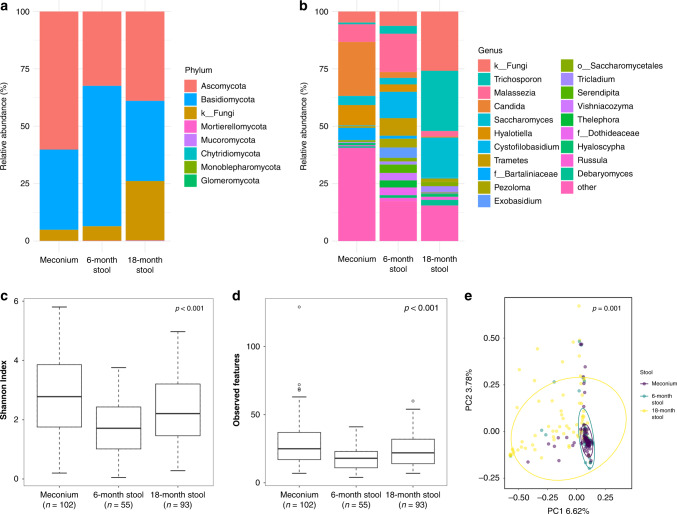
Taxonomic figures, alpha diversity, and beta diversity of all the stool samples by age at sampling. **a** All the phyla present in the samples for each time point. **b** The 20 most common genera in the samples for each time point. The rest of the genera are collapsed in the “other” group. **c** Shannon Index, using Kruskal–Wallis *H* as the statistical test. **d** Observed features, using Kruskal–Wallis *H* as the statistical test. **e** Bray–Curtis Dissimilarity between samples based on the sample age group. PERMANOVA was used as the statistical test for beta diversity.

We compared the fungal taxonomies at three time points: at birth (first-pass meconium), at 6 months of age, and at 18 months of age. The most abundant phyla in the meconium samples were Ascomycota (60%) and Basidiomycota (35%) (Fig. 1a and Supplementary Information 1), while the most common genera were *Candida* (24%)*, Hyalotiella* (8.9%), and *Malassezia* (7.7%) (Fig. 1b and Supplementary Information 1). At the age of 6 months, Basidiomycota was the most common phylum (61%), followed by Ascomycota (32%) (Fig. 1a and Supplementary Information 1), and *Candida* did not appear as the most abundant genus any longer, being replaced by *Malassezia* (17%), *Cystofilobasidium* (11%), *Trametes* (7.7%), and others (Fig. 1b and Supplementary Information 1). At the age of 18 months, Ascomycota and Basidiomycota were still the most abundant phyla (39% and 35%, respectively), but unidentified fungi had risen in prevalence (26%) (Fig. 1a and Supplementary Information 1). *Trichosporon* became the most abundant taxon (26%), followed by unidentified fungi (26%) and *Saccharomyces* (17%) (Fig. 1b and Supplementary Information 1). ANCOM analysis showed significant temporal changes in Ascomycota and Basidiomycota abundances from birth to the age of 18 months (Supplementary Information 2), and a total of 29 fungal genera showed differential abundances between the meconium, the 6-month stool and the 18-month stool (Supplementary Information 2).

The alpha diversity also showed significant differences between the three time points. The meconium samples showed the most diversity in terms of both the Shannon Index and observed features, while there was a drop in diversity in the 6-month samples followed by a rise in the 18-month samples (Fig. 1c, d). In the case of beta diversity, however, the samples obtained at birth and at the age of 6 months were clustered together, whereas the 18-month stool samples showed more diversity (Fig. 1e).

### Delivery mode affects the gut mycobiome of newborns and infants

**Fig. 2:**
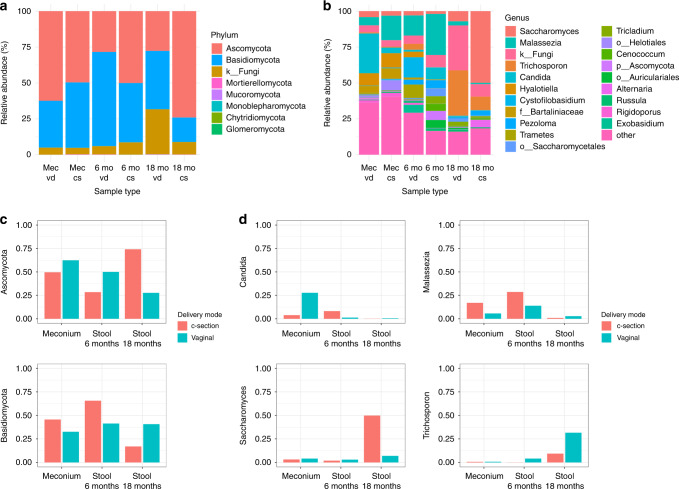
Taxonomic figures for each sample type based on the mode of delivery. **a** Relative abundances of the taxa at the phylum level. **b** The top 20 most abundant genera with the rest collapsed into the “other” group. **c** Changes in the abundance of Ascomycota (top) and Basidiomycota (bottom) over time in the vaginal delivery and Cesarean delivery groups. **d** Changes in the abundance of *Candida* (top left), *Malassezia* (top right), *Saccharomyces* (bottom left) and *Trichosporon* (bottom right) over time in the vaginal delivery and Cesarean delivery groups.

The stool samples differed at all three time points based on the delivery mode. In meconium samples, *Candida* was the most abundant genus in the vaginal samples (27%) and differed most notably between the vaginal and Cesarean delivery samples, whereas *Malassezia* (17%) was the most abundant genus in the Cesarean delivery samples (Fig. 2 and Supplementary Information 3). In 6-month stool samples, the most abundant genera were *Cystofilobasidium* (14%) and *Malassezia* (14%), while *Malassezia* remained the most abundant genus in the Cesarean delivery samples (29%) (Fig. 2 and Supplementary Information 3). In 18-month stool samples, the differences had grown, and the vaginal delivery samples mostly consisted of *Trichosporon* (32%) and unidentified fungi (31%), while the Cesarean delivery samples were dominated by *Saccharomyces* (50%) (Fig. 2 and Supplementary Information 3). The ANCOM and Mann–Whitney *U*-tests showed that differential abundances were found in 5 genera in the meconium, in 6 phyla and 3 genera in the 6-month stool samples, and 6 phyla and 4 genera in the 18-month stool samples (Supplementary Information 4).

**Fig. 3:**
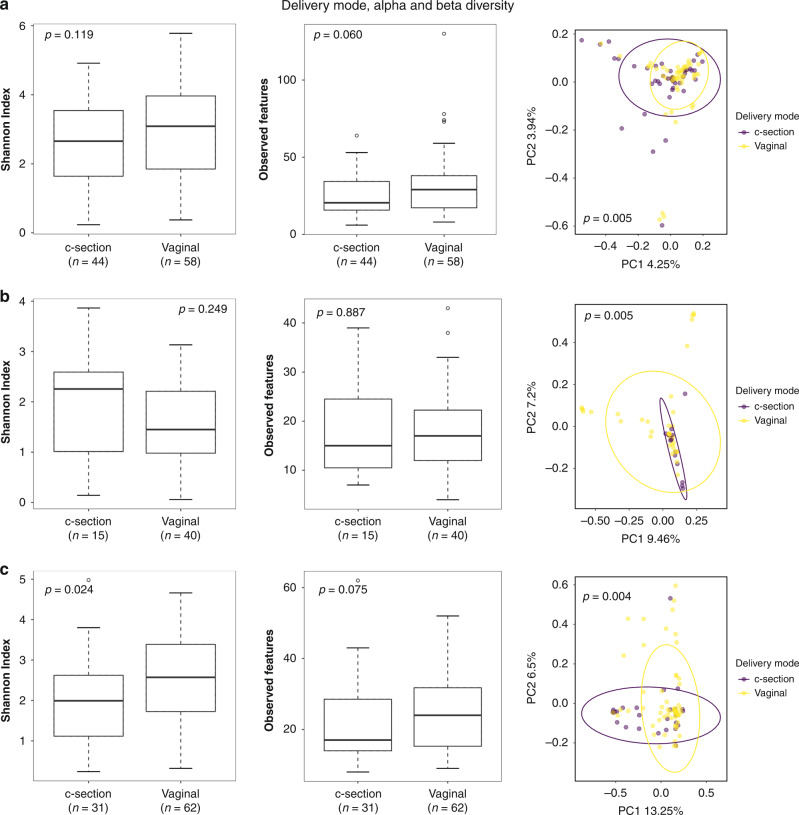
Alpha and beta diversity of all the samples by the mode of delivery. **a** Meconium, **b** 6-month stool, **c** 18-month stool. Alpha diversity metrics used: Shannon Index, observed features, with the Kruskal–Wallis *H* used as a statistical test. Beta diversity metric used: Bray–Curtis Dissimilarity, with PERMANOVA used as a statistical test. *p* values are shown in the respective figures.

No significant differences in alpha diversity were seen between the delivery modes until the age of 18 months (Fig. 3), but the beta diversity showed significant differences between the delivery modes at all three time points (Fig. 3). The Cesarean delivery samples showed higher diversity in meconium, but by 6 and 18 months the vaginal delivery samples had diversified more (Fig. 3).

### Intrapartum antibiotics affect the gut mycobiome in newborns and infants

**Fig. 4:**
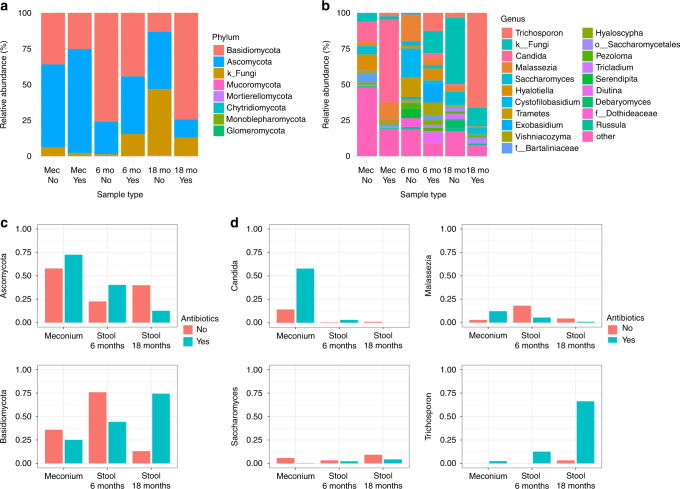
Taxonomic figures for each time point showing the effect of intrapartum antibiotic exposure in vaginal delivery samples. **a** Relative abundances of the taxa at the phylum level. **b** The top 20 most abundant genera with the rest collapsed into the “other” group. **c** Changes in the abundance of Ascomycota (top) and Basidiomycota (bottom) over time in the groups with and without intrapartum antibiotics. **d** Changes in the abundance of *Candida* (top left), *Malassezia* (top right), *Saccharomyces* (bottom left), and *Trichosporon* (bottom right) over time in the groups with and without intrapartum antibiotic exposure.

Differences based on intrapartum antibiotic usage were also found between the vaginal delivery samples. The meconium samples from the vaginal births with no intrapartum antibiotic exposure consisted of *Candida* (14%) and *Hyalotiella* (11%) as well as many other low-abundance taxa (44%), while in the vaginal samples exposed to intrapartum antibiotic treatment over half of the fungi consisted of *Candida* (58%), followed by *Malassezia* (12%) (Fig. 4 and Supplementary Information 5).

At the age of 6 months, the vaginal samples with no intrapartum antibiotic exposure had an even distribution of *Cystofilobasidium* (20%), *Malassezia* (18%), and *Trametes* (14%), and those with intrapartum antibiotic exposure had an even distribution of unidentified fungi (15%), *Exobasidium* (13%), and *Trichosporon* (13%) (Fig. 4 and Supplementary Information 5).

At the age of 18 months, the vaginal delivery samples with no intrapartum antibiotic treatment were dominated by unidentified fungi (46%), while those exposed to intrapartum antibiotics were dominated by *Trichosporon* (66%), followed by unidentified fungi (13%) (Fig. 4 and Supplementary Information 5). The ANCOM and Mann–Whitney *U*-tests showed differences in abundance in 3 genera in the meconium, 3 phyla and 5 genera in the 6-month stool samples, and 5 phyla and 1 genus in the 18-month stool samples (Supplementary Information 4).

**Fig. 5:**
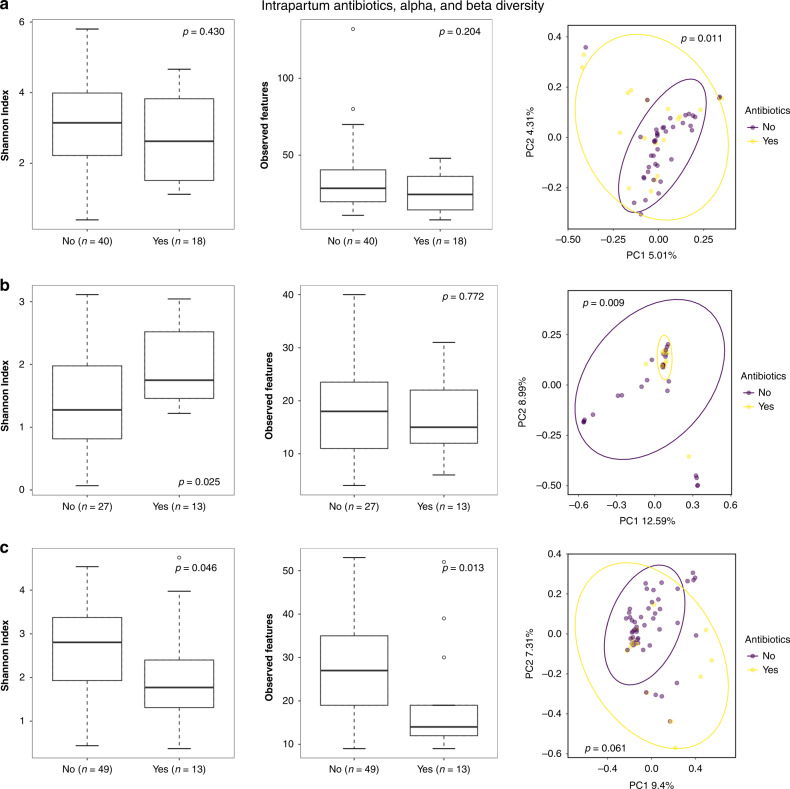
Alpha and beta diversity of vaginal delivery samples showing the effect of the intrapartum antibiotic exposure. **a** Meconium samples. **b** 6-month stool samples. **c** 18-month stool samples. Alpha diversity metrics used: Shannon Index, observed features, with Kruskal–Wallis *H* used as a statistical test. Beta diversity metric used: Bray–Curtis Dissimilarity, with PERMANOVA used as a statistical test. *p* values are shown in the respective figures.

When measuring alpha diversity in the vaginal samples based on the usage of intrapartum antibiotics, significant differences were found in the 6-month samples (Shannon Index *p* = 0.025) and 18-month samples (Shannon Index *p* = 0.046, observed features *p* = 0.013) (Fig. 5). In the case of beta diversity, on the other hand, intrapartum antibiotic exposure showed significantly greater diversity between samples in the meconium (*p* = 0.011), but at the age of 6 months the samples with no exposure to intrapartum antibiotics were more diverse (*p* = 0.009; Fig. 5). By 18 months, the effect of exposure to intrapartum antibiotics on beta diversity was no longer significant (*p* = 0.061; Fig. 5).

## Discussion

This prospective cohort study characterized the development of the gut fungal microbiome, or the gut mycobiome, from birth to the age of 18 months. Delivery mode and exposure to intrapartum antibiotic prophylaxis were markedly associated with gut mycobiome composition from birth to 18 months of age.

Previous studies have shown that perinatal antibiotic exposure changes the gut bacterial microbiome, or bacteriome, in children.^21,22,30^ Fungi and bacteria likely interact in complex ways in the gut microbiome, either through mutualism or competition.^31^ Interestingly, antibacterial agents administered at birth, without having any direct antimicrobial effect on fungi, appeared to influence the development of the gut mycobiome in vaginally delivered infants as well, possibly through interaction with the gut bacteriome.

We found here that the meconium was dominated by the genus *Candida*, which has been found earlier to colonize the gut,^32^ and has been shown to be the most common fungal colonizer in the infant gut mycobiome according to a Swedish longitudinal study of 133 infants.^33^ The abundance of *Candida* had dropped by the age of 6 months, however, and the abundance of *Malassezia* and *Cystofilobasidium* had risen. *Malassezia* has been found to be a common gut colonizer, shifts in the abundance of which have previously been associated with IBD and IBS,^10,34^ and *Cystofilobasidium* has also been identified in the gut mycobiome of young children.^18^

At the age of 18 months, the stool samples were dominated by the genus *Trichosporon*, unidentified fungi and *Saccharomyces. Trichosporon* has been found to be associated with various infections in humans, and specifically *T. asahii* has been recognized as a common gut colonizer.^35^ *Saccharomyces* is a genus that includes many yeasts, and the species *S. boulardii* is known to act as a probiotic in the gut mycobiome.^36^ We found high abundances of the phyla Ascomycota and Basidiomycota from birth to 18 months of age. Phyla Ascomycota and Basidiomycota have previously been reported as dominant phyla colonizing the human gut, the ratio between which has been reported to affect the prevalence of IBD.^34^

The mode of delivery has been found previously to affect the bacterial composition of the human gut,^37–41^ and our results show in addition that it can markedly affect the newborn and infant gut mycobiome. Infants born by the vaginal route had the highest amounts of the genus *Candida* in their gut mycobiome at birth, whereas those born via Cesarean delivery had a much lower abundance of *Candida* in their meconium samples. *Candida* is a common yeast pathogen and colonizer of the vagina,^42^ and the present results suggest that vaginal *Candida* is likely to be one of the main sources of initial gut mycobiome development in vaginally delivered newborns. Interestingly, however, the effect of delivery mode on the gut mycobiome seemed to remain detectable until the age of 18 months of life, suggesting possible long-term effects.

Given that exposure to antibiotics at birth has previously been shown to influence the development of the gut bacteriome,^21,22,30,43^ we found that intrapartum antibacterial antibiotics, without having any direct effect on fungi, significantly affected the gut mycobiome of vaginally born newborns for at least 18 months after birth. Exposure to antibacterial agents has previously been shown to have a long-term influence on the human gut mycobiome in a cohort of 14 healthy adults, possibly by influencing bacterial-fungal interactions, including disruption and resilience of fungal community compositions.^31^ Our analogous finding was that antibiotic exposure at birth altered the development of the gut mycobiome at least up to 18 months of age, most likely by influencing bacterial–fungal interactions.

There are still limited data available on the role of the mycobiome in the human gut microbiome because most existing observations have been focused on the bacteriome. This is understandable, since the human gut microbiome has been estimated to consist of 99% bacteria.^44^ The role of other microbes may also be important, however, either through direct host–microbe interactions, or alternatively, through microbe–microbe interactions. This has been demonstrated by specifically a gut commensal *Candida albicans*, which has been shown to interact with gut bacteria and cause both bacterial and fungal dysbiosis.^45^ Environmental effects such as diet and the use of antibiotics have already been observed to affect the mycobiome and bacteriome alike, which may be partly caused by the mutualistic relationship between fungi and bacteria.^46–48^

The strength of our work is that it is one of the first prospective cohort studies comparing the effect of delivery mode and intrapartum antibiotic exposure on the development of the gut mycobiome in infants and young children. Furthermore, we had a sizeable cohort which enabled us to make reliable and meaningful comparisons. We also included a large negative control group in order to identify and filter out possible contaminant fungi present in the reagents, since the effect of contaminant DNA, especially on low biomass samples, has been recognized as an important factor to consider during the analysis of bacteriome findings.^26^ One limitation of the present study is that we cannot present exact taxonomic data on the fungi. Although UNITE is one of the most comprehensive fungal databases, it still lacks data on the lower taxonomic levels, especially the genus and species levels, when it comes to characterizing the gut mycobiome. Furthermore, like bacteriome studies,^49^ mycobiome studies suffer from primer bias, as the ITS and 18S genes that are used as fungal identifiers yield varying results.^50^ As there is no clear consensus on which gene to target for mycobiome analysis, a combination of both might yield the most accurate results.

## Conclusion

In conclusion, both delivery mode and antibacterial agents could be shown to alter the development of the gut mycobiome in this prospective study of infants and young children. As the gut mycobiome composition has previously been associated with human health,^8–16^ the present findings suggest that the fungal microbiome is likely to be relevant when investigating the role of perinatal factors and the gut microbiome in health.

## Supplementary information

Supplementary Information

## Data Availability

The raw sequences were uploaded in BioProject with the accession number PRJNA831656.
