# Supplementary material for: Development of gut mycobiome in infants and young children: a prospective cohort study
Source: Pediatr Res. 2023 Jan 20;94(2):486–94. doi: 10.1038/s41390-023-02471-y (PMC10382308; doi:10.1038/s41390-023-02471-y)
Supplement: Supplementary file 1 — Supplementary Information [file 41390_2023_2471_MOESM1_ESM.pdf]

Meconium		Stool 6 months		Stool 18 months	
**Phylum**	**%**	**Phylum**	**%**	**Phylum**	**%**
Ascomycota	60	Basidiomycota	61	Ascomycota	39
Basidiomycota	35	Ascomycota	32	Basidiomycota	35
k. Fungi	4.8	k. Fungi	6.3	k. Fungi	26
Mortierellomycota	0.034	Mortierellomycota	0.15	Mucoromycota	0.17
Mucoromycota	0.027	Monoblepharomycota	0.007	Mortierellomycota	0.078
Chytridiomycota	0.013	Mucoromycota	0.001		
**Genus**	**%**	**Genus**	**%**	**Genus**	**%**
Candida	24	*Malassezia*	17	*Trichosporon*	26
Hyalotiella	8.9	*Cystofilobasidium*	11	k. Fungi	26
Malassezia	7.7	*Trametes*	7.7	*Saccharomyces*	17
f. Bartaliniaceae	5.3	k. Fungi	6.3	*Tricladium*	2.5
k. Fungi	4.8	Exobasidium	4.5	*Debaryomyces*	2.5
Saccharomyces	4.0	*Pezoloma*	3.8	*Malassezia*	2.4
o. Helotiales	2.4	*Serendipita*	3.7	o. Saccharomycetales	1.9
Fomitopsis	1.9	*Trichosporon*	3.4	*Fusarium*	1.5
Rigidoporus	1.8	f. Dothideaceae	3.4	*Pezoloma*	1.5
Trichoderma	1.6	*Vishniacozyma*	3.2	*Alternaria*	1.4
Other	38	Other	36	Other	17

Supplementary information 1. The most abundant fungal taxa in each sample type (meconium, 6-month stool, 18-month stool). The ten most abundant taxa are included at the genus level, and the rest make up the "other" category.

Phylum	ANCOM	Genus	ANCOM
k_Fungi	7	f_Bartaliniaceae	430
Ascomycota	7	*Hyalotiella*	430
Basidiomycota	7	k_Fungi	429
		Saccharomyces	429
		Trichosporon	429
		Fomitopsis	425
		Tricladium	423
		Rhodotorula	423
		Rigidoporus	422
		Wilcoxina	421
		Pezoloma	420
		o_Helotiales	418
		Thelebolus	418
		Trichoderma	416
		Cenococcum	410
		Mycena	409
		Hyaloscypha	408
		o_Saccharomycetales	405
		Candida	404
		Phialocephala	398
		Hypholoma	395
		f_Pleosporaceae	395
		Ganoderma	394
		Filobasidium	394
		Ramularia	394
		Cortinarius	385
		Coprinellus	383
		Neosetophoma	376
		Trechispora	366

Supplementary information 2. ANCOM (W) of all samples based on the age of the stool at the phylum and genus levels.

**Vaginal delivery, meconium samples**				**C-section delivery, meconium samples**			
**Phylum (%)**		**Genus (%)**		**Phylum (%)**		**Genus (%)**	
Ascomycota	62	*Candida*	28	Ascomycota	50	*Malassezia*	17
Basidiomycota	33	*Hyalotiella*	8.6	Basidiomycota	46	*Hyalotiella*	10
k. Fungi	4.9	*Malassezia*	5.7	k. Fungi	4.5	f. Bartaliniaceae	7.3
Chytridiomycota	0.016	f. Bartaliniaceae	4.9	Mortierellomycota	0.19	o. Helotiales	6.9
Mucoromycota	0.015	k. Fungi	4.9	Mucoromycota	0.082	k. Fungi	4.5
Mortierellomycota	0.001	*Saccharomyces*	4.1			*Naganishia*	4.2
		Fomitopsis	1.9			*Candida*	3.9
		Trichoderma	1.7			*Saccharomyces*	3.2
		Rigidoporus	1.7			*Rigidoporus*	2.6
		o. Helotiales	1.5			*Rhodotorula*	2.4
		other	37			other	38
**Vaginal delivery, 6-month stool samples**				**C-section delivery, 6-month stool samples**			
**Phylum (%)**		**Genus (%)**		**Phylum (%)**		**Genus (%)**	
Basidiomycota	66	*Cystofilobasidium*	14	Ascomycota	50	*Malassezia*	29
Ascomycota	28	*Malassezia*	14	Basidiomycota	41	k. Fungi	8.6
k. Fungi	5.8	*Trametes*	9.4	k. Fungi	8.6	*Candida*	8.2
Mortierellomycota	0.18	k. Fungi	5.8	Monoblepharomycota	0.039	p. Ascomycota	6.3
Mucoromycota	0.001	*Exobasidium*	5.5			Pezoloma	5.6
		Serendipita	4.5			o. Auriculariales	5.5
		Trichosporon	4.1			o. Saccharomycetales	5.5
		f. Dothideaceae	4.1			*Tricladium*	5.2
		Vishniacozyma	4.0			*Cenococcum*	5.0
		Hyalotiella	4.0			*Pseudeurotium*	3.8
		other	31			other	18
**Vaginal delivery, 18-month stool samples**				**C-section delivery, 18-month stool samples**			
**Phylum (%)**		**Genus (%)**		**Phylum (%)**		**Genus (%)**	
Basidiomycota	41	*Trichosporon*	32	Ascomycota	74	*Saccharomyces*	50
k. Fungi	31	k. Fungi	31	Basidiomycota	17	*Trichosporon*	9.4
Ascomycota	28	*Saccharomyces*	7.1	k. Fungi	8.7	k. Fungi	8.7
Mucoromycota	0.22	*Debaryomyces*	3.3	Mortierellomycota	0.14	*Alternaria*	5.1
Mortierellomycota	0.059	*Tricladium*	3.0	Mucoromycota	0.013	*Cyberlindnera*	4.0
		Malassezia	2.8	Glomeromycota	0.001	*Pezoloma*	3.3
		o. Saccharomycetales	2.3			*Phialocephala*	3.2
		Fusarium	2.0			*Ramularia*	2.1
		Russula	1.5			f. Thelephoraceae	2.0
		Hyaloscypha	1.4			*Thelephora*	1.3
		other	14			other	11

Supplementary information 3. The most abundant fungal taxa at each time point (meconium, 6-month stool, 18-month stool) by mode of delivery. The ten most abundant taxa are included at the genus level and the rest make up the “other” category.

**Intrapartum antibiotics (vaginal samples)**			**Delivery mode**		
**Meconium**					
	**ANCOM**	**Mann-Whitney U**		**ANCOM**	**Mann-Whitney U**
**Genus**			**Genus**		
Coprinellus	245		*Cutaneotrichosporon*	81	<0.001
Rigidoporus	229	0.003	*Coprinellus*		<0.001
Cutaneotrichosporon		0.023	*Gymnopus*		0.006
			Rigidoporus		0.012
			Serendipita		0.011
**6-month stool**					
	**ANCOM**	**Mann-Whitney U**		**ANCOM**	**Mann-Whitney U**
**Phylum**			**Phylum**		
Basidiomycota	2	0.015	Ascomycota	1	<0.001
k_Fungi	1	0.007	Basidiomycota	1	0.03
Ascomycota	1		k_Fungi	0	
			Monoblepharomycota	0	
			Mortierellomycota	0	
			Mucoromycota	0	
**Genus**			**Genus**		
Candida		0.004	*Candida*		0.029
Trichosporon		<0.001	*Trichosporon*		0.013
Rigidoporus		0.034	*Cystofilobasidium*		0.030
Cutaneotrichosporon		0.023			
Cryptococcus		0.005			
**18-month stool**					
	**ANCOM**	**Mann-Whitney U**		**ANCOM**	**Mann-Whitney U**
**Phylum**			**Phylum**		
k_Fungi	3		k_Fungi	3	
Ascomycota	3		Ascomycota	3	
Basidiomycota	3		Basidiomycota	3	
Glomeromycota	3		Glomeromycota	3	
Mortierellomycota	3		Mortierellomycota	3	
			Mucoromycota	3	
**Genus**			**Genus**		
k_Fungi	228		*Trichosporon*		0.0506
			Bartaliniaceae		0.05179
			Cystofilobasidium		0.019
			Cryptococcus		0.019

Supplementary information 4. ANCOM (W) and Mann-Whitney U (p-value) results for the differences associated with the use of intrapartum antibiotics and the choice of delivery mode in all samples at the phylum and genus levels, quoted separately for the meconium, 6-month stools and 18-month stools.

Vaginal delivery, meconium, no intrapartum antibiotics				Vaginal delivery, meconium, intrapartum antibiotics			
**Phylum (%)**		**Genus (%)**		**Phylum (%)**		**Genus (%)**	
Ascomycota	58	*Candida*	14	Ascomycota	73	*Candida*	58
Basidiomycota	36	*Hyalotiella*	11	Basidiomycota	25	*Malassezia*	12
k. Fungi	6.1	f. Bartaliniaceae	6.6	k. Fungi	2.2	f. Pleosporaceae	4.7
Mucoromycota	0.021	k. Fungi	6.1	Chytridiomycota	0.053	*Hyalotiella*	2.8
Mortierellomycota	0.001	*Saccharomyces*	5.8	Mucoromycota	0.002	*Trichosporon*	2.4
		Malassezia	2.8	Mortierellomycota	0.002	k. Fungi	2.2
		Trichoderma	2.3			f. Bartaliniaceae	1.1
		Fomitopsis	2.3			*Fomitopsis*	1.0
		Rigidoporus	2.2			*Thelebolus*	0.72
		o. Helotiales	1.9			*Russula*	0.66
		other	44			other	15

Vaginal delivery, 6-month stool, no intrapartum antibiotics				Vaginal delivery, 6-month stool, intrapartum antibiotics			
**Phylum (%)**		**Genus (%)**		**Phylum (%)**		**Genus (%)**	
Basidiomycota	76	*Cystofilobasidium*	20	Basidiomycota	44	k. Fungi	15
Ascomycota	23	*Malassezia*	18	Ascomycota	40	*Exobasidium*	13
k. Fungi	1.1	*Trametes*	14	k. Fungi	15	*Trichosporon*	13
Mortierellomycota	0.27	*Serendipita*	6.6			*Vishniacozyma*	9.3
Mucoromycota	0.001	f. Dothideaceae	6.1			*Hyalotiella*	8.4
		Thelephora	5.6			*Diutina*	6.8
		Pezoloma	4.0			*Malassezia*	5.5
		Saccharomyces	3.3			*Hyaloscypha*	3.3
		Rigidoporus	2.0			*Candida*	3.1
		Russula	1.9			f. Bartaliniaceae	3.0
		other	19			other	19

Vaginal delivery, 18-month stool, no intrapartum antibiotics				Vaginal delivery, 18-month stool, intrapartum antibiotics			
**Phylum (%)**		**Genus (%)**		**Phylum (%)**		**Genus (%)**	
k. Fungi	46	k. Fungi	46	Basidiomycota	74	*Trichosporon*	66
Ascomycota	40	*Saccharomyces*	9.3	k. Fungi	13	k. Fungi	13
Basidiomycota	13	*Debaryomyces*	5.9	Ascomycota	13	*Saccharomyces*	4.3
Mucoromycota	0.40	*Malassezia*	4.4			*Cortinarius*	2.5
Mortierellomycota	0.11	*Tricladium*	3.9			o. Saccharomycetales	2.1
		Fusarium	3.6			*Hyaloscypha*	2.1
		Trichosporon	3.3			o. Auriculariales	2.1
		o. Saccharomycetales	2.5			*Tricladium*	1.8
		Russula	1.8			*Russula*	1.2
		Wilcoxina	1.8			p. Ascomycota	1.1
		other	17			other	3.6

Supplementary information 5. The effect of intrapartum antibiotic exposure on the most abundant fungal taxa at each time point (meconium, 6-month stool, 18-month stool). The ten most abundant taxa have been included at the genus level, and the rest of the taxa make up the “other” category.
